# Nitroacetylene as dipolarophile in [2 + 3] cycloaddition reactions with allenyl-type three-atom components: DFT computational study

**DOI:** 10.1007/s00706-014-1389-0

**Published:** 2015-01-27

**Authors:** Radomir Jasiński

**Affiliations:** Institute of Organic Chemistry and Technology, Cracow University of Technology, Cracow, Poland

**Keywords:** Cycloaddition, Mechanism, Regioselectivity, DFT study

## Abstract

**Abstract:**

[2 + 3] Cycloaddition reactions of nitroacetylene with allenyl-type three-atom components take place according to the polar, but a one-step mechanism. Alternatively to cycloadducts, during the reaction between the aforementioned reagents, zwitterionic structures with “extended” conformation may be formally created. However, this route is supported by neither kinetic nor thermodynamic factors.

**Graphical Abstract:**

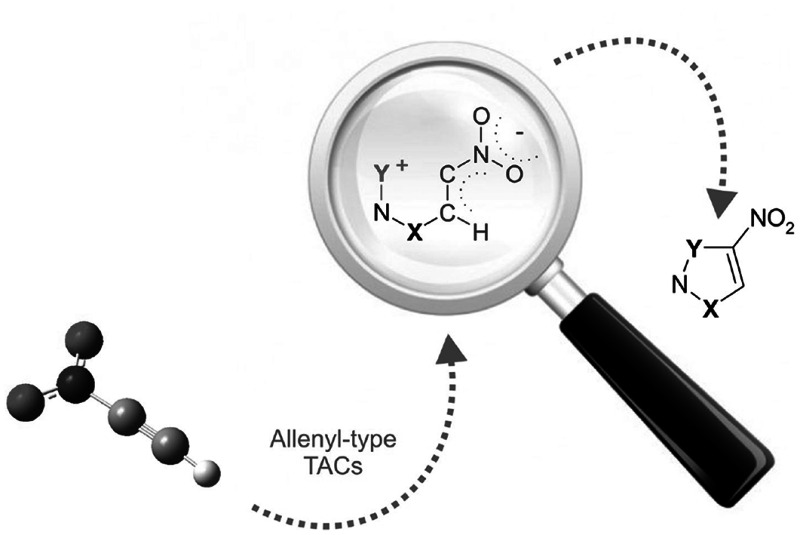

## Introduction

Nitroacetylenes belong—because of the presence of a strongly electron withdrawing NO_2_-group—to a class of strongly electrophilic acetylenes. They are relatively unstable, and their physicochemistry is very poorly known [1].

In particular, very little is known about the simple representative of this group of compounds—parent nitroacetylene (**1**). Recently, the preliminary results of experimental studies related to nitroacetylene as a Diels–Alder reaction component were published [2]. However, no systematic studies of its participation in reactions of [2 + 3] cycloaddition have been performed to date. This work initiates research in this area; in particular, within the study, complex, quantum-chemical studies of the [2 + 3] cycloaddition reaction of nitroacetylene (**1**) to selected allenyl-type three-atom components (TACs). Three such TACs were selected, often tested in recent years as components of [2 + 3] cycloaddition reactions with electrophilic ethylenes: benzonitrile *N*-oxide (**2a**) [3–6], phenyl azide (**2b**) [6–8], and phenyldiazomethane (**2c**) [6, 9] (Scheme 1).

**Figure:**
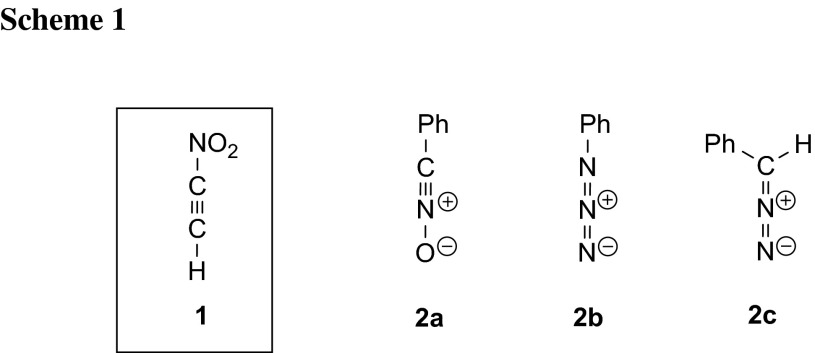

Reactions of these TACs with nitroacetylene are a potentially highly effective and selective method of synthesis of nitro-substituted, five-membered, unsaturated heterocycles that are valuable from the point of view of organic preparatory work [10–12]. For this reason, identification of factors determining their course is so important. With this in mind, the following work was performed: (1) an analysis of nature of reagent interactions based on reactivity indices theory, and (2) simulations of a theoretically possible reaction path in the presence of a weakly polar (toluene), and strongly polar medium (nitromethane). It must be stressed here that the problematics of the [2 + 3] cycloaddition reaction of the aforementioned reagents is also very interesting from a mechanistic point of view. These reactions may also take place according to a one-step mechanism, as well as to a two-step mechanism, with a zwitterionic intermediate [13–15]. The two-step mechanism is facilitated in this case by (1) the strongly electrophilic character of the dipolarophile and the nucleophilic nature of the TACs and (2) unequal screening of the reaction centres of both reagents.

## Results and discussion

Nitroacetylene (**1**) is characterized by high global electrophilicity [16, 17], exceeding 3 eV. Using the scale proposed by Domingo [17], it should be classified as belonging to the group of strong electrophiles. On the other hand, TACs **2a**–**2c** have a significantly weaker electrophilic nature (*ω* < 1.6 eV)—using the aforementioned scale they are classified as moderate electrophiles. Thus, they should behave as nucleophiles in reactions with nitroacetylene. The nucleophilic character of these compounds is indicated by values of the *N* indices. As can be concluded from the data summarized in Table 1, phenzyldiazomethane (**2c**) is the strongest nucleophile in the analysed series, while benzonitrile *N*-oxide (**2a**) is the weakest nucleophile. It should be noted that in cases of all reagent pairs, the difference in global electrophilicities exceeds 1.5 V. The title reactions are thus polar processes [18].

**Table 1:** Essential electronic properties of nitroacetylene (**1**) and TACs **2a**–**2c**

	Global properties			Local properties							
	*μ*/a.u.	*ω*/eV	*N*/eV	*P*_X_^−^	*P*_Y_^−^	*N*_X_/eV	*N*_Y_/eV	*P*_α_^+^	*P*_β_^+^	*ω*_α_/eV	*ω*_β_/eV
**1**	−0.1771	3.14						−0.10	0.33	−0.30	1.05
**2a**	−0.1406	1.46	2.78	0.42	0.01	1.18	0.03				
**2b**	−0.1330	1.27	2.92	0.21	0.19	0.60	0.57				
**2c**	−0.1276	1.55	3.71	0.34	0.25	1.26	0.94				

Next, it was decided to determine which of the theoretically possible directions of substrate transformations would be favoured by the electrophile–nucleophile interactions. In the case of every single one of the cycloaddition reactions studied, two regioisomeric reaction paths can be studied (Scheme 2A, B).

**Figure:**
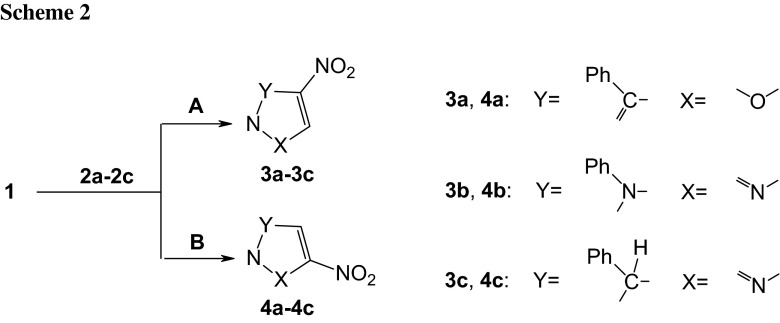

Analysis of local electronic properties allows one to state that the most electrophilically activated centre of the nitroacetylene molecule is the C_β_ carbon atom. Local electrophilicity on the C_α_ carbon atom is more than three times weaker. On the other hand, the terminal X atom of TACs is the most nucleophilic activated centre in these species. If it is assumed that the reaction route is determined by an attack of the more strongly nucleophilic reaction centre of the TAC on the more strongly electrophilic reaction centre of nitroacetylene, then the favoured direction of reagent transformation should always be determined by the **A** path (see Scheme 2).

### Reaction profiles

Taking into account the nature of the electrophile–nucleophile interactions, reactions of nitroacetylene with TACs **2a**–**2c** must be recognized as polar processes. This, however, does not determine their mechanism. There are two possible variants of the conversion of reagents into adducts: (a) a one-step polar mechanism and (b) two-step zwitterionic mechanism. In the first case, one should expect a transition state (TS) in the energy profile, in the second two TSs connected by a valley corresponding to the zwitterionic intermediate (respectively, **5** or **6**, see Scheme 2).

As suggested by quantum-chemical calculations, in weakly polar toluene (*ε* = 2.38), the reaction of nitroacetylene with benzonitrile *N*-oxide takes place—regardless of the regioisomeric path—as a one-step process. All attempts at finding paths leading to zwitterionic structures ended unsuccessfully.

Formation of a pre-reaction complex (local minimum—LM) always comprises the first reaction step. This is related to a certain drop in enthalpy of the reacting system. It should be noted, that LMs are exclusively enthalpic in character because Gibbs free energy gap between suitable intermediate and reactants is always greater than zero (Δ*G* > 0) due to the entropic factor (*T*Δ*S*). Therefore, pre-reaction complexes may not exist as stable products.

Only then does the system start heading towards the activation barrier. By analysing barrier heights on paths **A** and **B**, it must be said that both regioisomeric substrate transformation routes are possible from the kinetic point of view. However, the favoured path is the one leading finally to 4-nitroisoxazole (**3a**). This conclusion is in agreement with forecasts based on reactivity indices analysis.

**Fig. 1:**
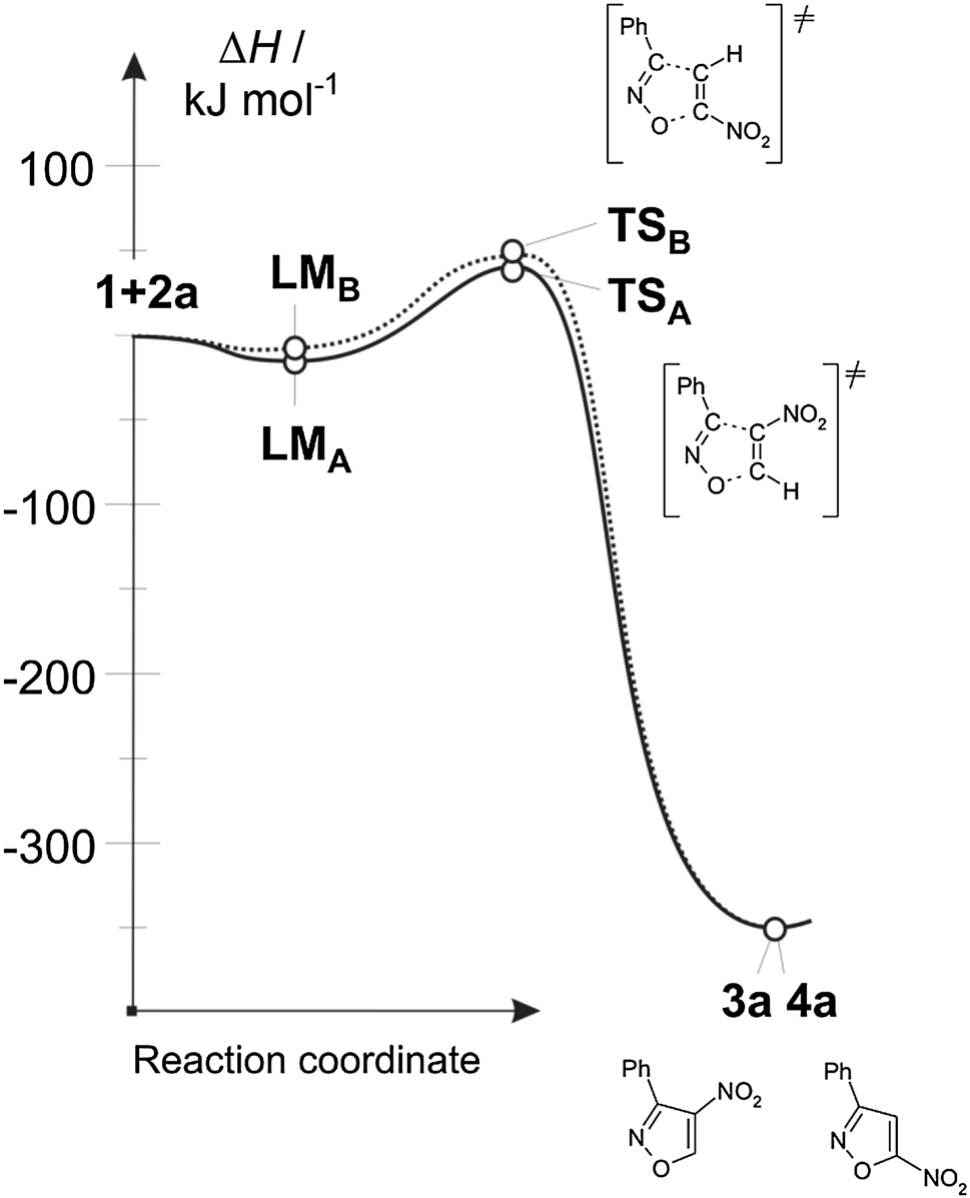
Enthalpy profiles for reaction between nitroacetylene (**1**) and benzonitrile *N*-oxide (**2a**) in toluene according to DFT calculations at B3LYP/6-31G(d) theory level

A similar picture of **1** **+** **2a** reaction is provided by calculations at more advanced levels of theory B3LYP/6-31+G(d), B3LYP/6-311G(d), and B3LYP/6-311+G(d) (Fig. 1).

DFT calculations also indicate a one-step mechanism of cycloaddition reaction of nitroacetylene to TACs **2b** and **2c**. The performed simulations suggest, however, a different reaction regioselectivity than would result from reactivity index analysis. In particular, in the case of cycloaddition reactions using phenyl azide (**2b**), both regioisomeric reaction channels should be permitted, but path **B** will be favoured. However, in the case of a cycloaddition reaction using phenyldiazomethane, path **A** must be treated as kinetically forbidden. DFT calculations indicate that the only permitted cycloaddition channel is the path leading to the **4c** adduct.

**Fig. 2:**
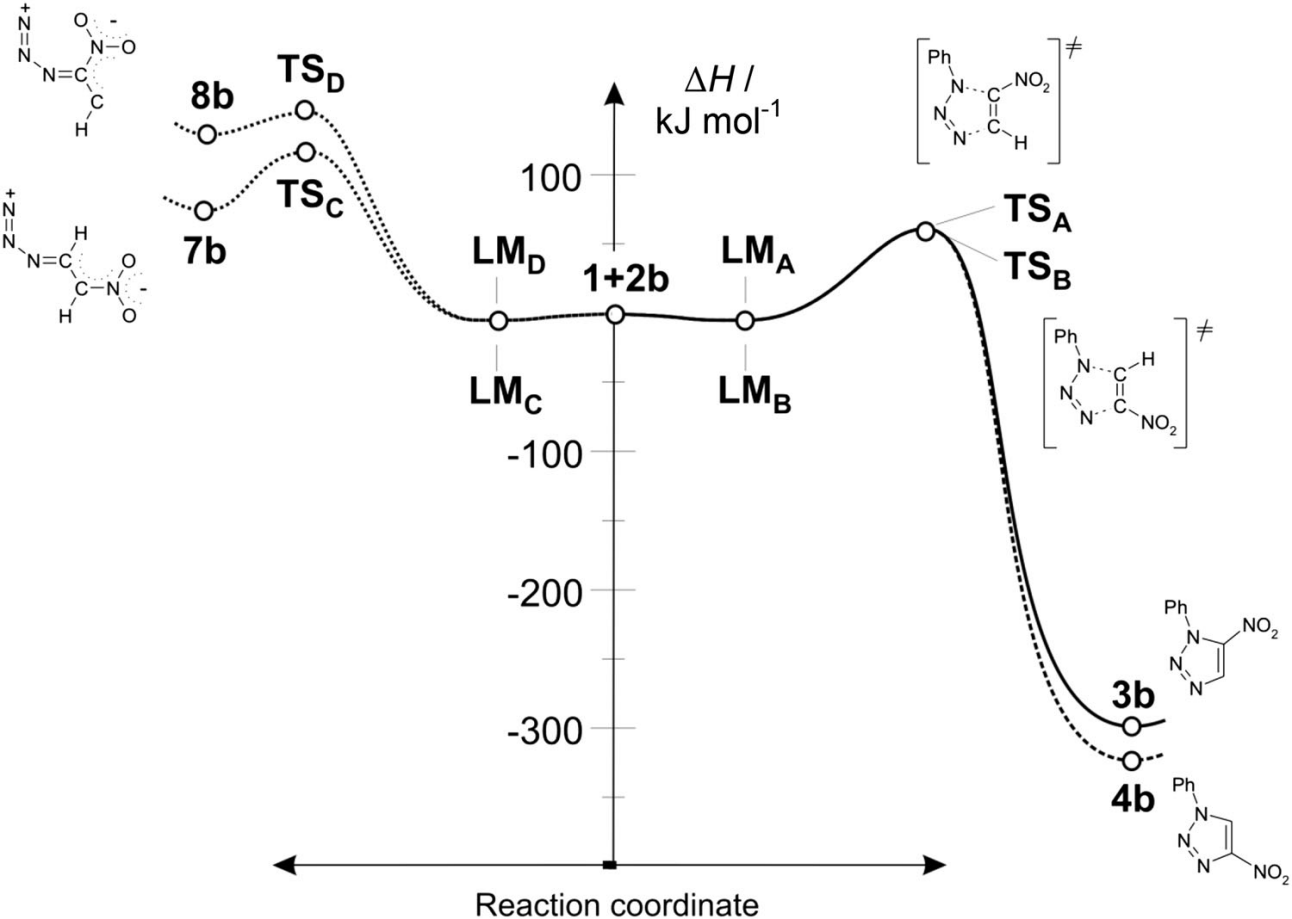
Enthalpy profiles for reaction between nitroacetylene (**1**) and phenylazide (**2b**) in toluene according to DFT calculations at B3LYP/6-31G(d) theory level

Analysing conversion routes for reagent pairs **1** + **2b**, paths leading to zwitterionic structures were found (Fig. 2). However, these are not the expected zwitterions **5** and **6** with “cyclic” conformation (see. Scheme 3) but zwitterions with “extended” conformation **7** and **8** (see Scheme 4). Their conversion to adducts can be executed via a step of dissociation into individual reagents and (in the next step) a stage of cycloaddition according to a one-step mechanism. These paths should be treated as formally forbidden from the kinetic point of view. Thermodynamic factors also do not favour formation of zwitterions **7** and **8**.

**Figure:**
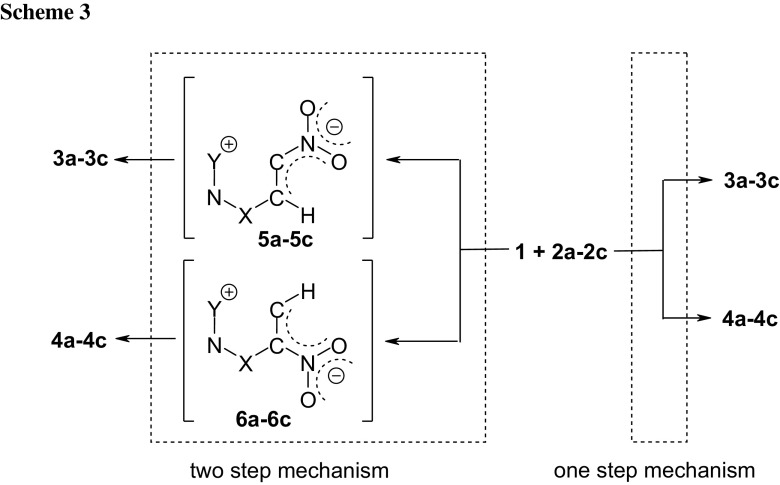

**Figure:**
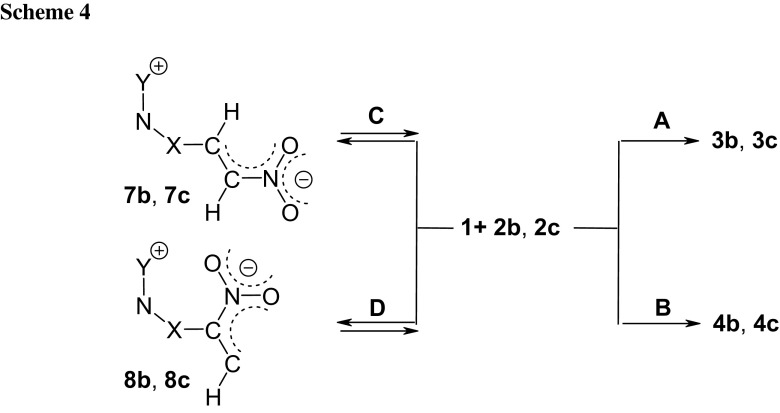

Introduction of a more polar medium of nitromethane (*ε* = 38.20) into the reaction mixture does not result in qualitative changes in energy profiles of the reaction. However, their quantitative description does change. In particular, activation barriers in cases of relatively less polar cycloaddition reactions **1** **+** **2a** → **3a**/**4a** and **1** **+** **2b** → **3b**/**4b** increase. In case of the most polar of the considered cycloaddition reactions (**1** **+** **2c** → **3c**/**4c**), activation barriers become slightly lower in a more polar medium. However, in the case of all reactions leading to zwitterions **7** and **8** an increase in the polarity of the reaction medium facilitates the lowering of the activation barrier. However, this lowering is not significant enough to treat these processes as permitted kinetically.

### Key structures

Within the area of located pre-reaction complexes **LM** distances between reaction centres still remain far outside the range typical for σ bonds in intermediate states. Some **LM**s are orientation complexes. None of them has, however—regardless of reaction medium polarity—the nature of a charge-transfer complex (CT) [see global electron density transfer indices (GEDT) in Table 2].

**Table 2:** Selected properties of critical structures for reactions between nitroacetylene (**1**) and TACs **2a**–**2c** according to B3LYP/6-31G(d) calculations

Solvent	Reaction	Structure	Interatomic distances								∆*l*	*GEDT*/*e*
			X-C_α_		X-C_β_		Y-C_α_		Y-C_β_			
			*r*/Ǻ	*l*	*r*/Ǻ	*l*	*r*/Ǻ	*l*	*r*/Ǻ	*l*		
Toluene (*ε* = 2.38)	**1** + **2a**	**LM**_**A**_			3.103		4.246					
		**TS**_**A**_			2.130	0.392	2.392	0.340			0.05	0.11
		**3a**			1.325		1.441					
		**LM**_**B**_	3.328						5.598			
		**TS**_**B**_	2.321	0.248					2.219	0.460	0.21	0.11
		**4a**	1.325						1.441			
	**1** + **2b**	**LM**_**A**_			1.441		1.441					
		**TS**_**A**_			2.117	0.433	2.241	0.361			0.07	0.05
		**3b**			1.351		1.367					
		**LM**_**B**_	4.223						7.214			
		**TS**_**B**_	2.279	0.317					2.131	0.421	0.10	0.08
		**4b**	1.354						1.350			
		**LM**_**C**_			4.741							
		**TS**_**C**_			1.728	0.699						0.26
		**7b**			1.329							
		**LM**_**D**_	3.507									
		**TS**_**D**_	1.549	0.880								0.21
		**8b**	1.383									
	**1** + **2c**	**LM**_**A**_			4.106		3.872					
		**TS**_**A**_			2.285	0.401	2.335	0.438			0.04	0.20
		**3c**			1.429		1.495					
		**TS**_**B**_	2.513	0.237					2.220	0.513	0.28	0.21
		**4c**	1.426						1.493			
		**TS**_**C**_			1.845	0.619						0.28
		**7c**			1.336							
		**TS**_**D**_	1.623	0.798								0.36
		**8c**	1.350									
Nitromethane (*ε* = 38.20)	**1** + **2a**	**LM**_**A**_			3.122		4.287					
		**TS**_**A**_			2.106	0.410	2.409	0.326			0.08	0.13
		**3a**			1.324		1.439					
		**LM**_**B**_	3.396						5.636			
		**TS**_**B**_	2.320	0.268					2.204	0.459	0.19	0.12
		**4a**	1.339						1.431			
	**1** + **2b**	**LM**_**A**_			5.770		4.195					
		**TS**_**A**_			2.103	0.443	2.248	0.354			0.09	0.07
		**3b**			1.351		1.366					
		**LM**_**B**_	3.306						5.770			
		**TS**_**B**_	2.312	0.294					2.106	0.438	0.14	0.10
		**4b**	1.355						1.348			
		**LM**_**C**_			4.675							
		**TS**_**C**_			1.777	0.702						0.29
		**7b**			1.369							0.29
		**LM**_**D**_	3.615									
		**TS**_**D**_	1.561	0.880								0.33
		**8b**	1.394									0.32
	**1** + **2c**	**LM**_**A**_			5.441		8.076					
		**TS**_**A**_			2.219	0.446	2.388	0.401			0.04	0.21
		**3c**			1.428		1.494					
		**TS**_**B**_	2.596	0.176					2.200	0.526	0.35	0.27
		**4c**	1.424						1.492			
		**TS**_**C**_			1.915	0.585						0.30
		**7c**			1.353							0.25
		**TS**_**D**_	1.621	0.819								0.40
		**8c**	1.373									0.31

New σ bonds are formed after the system reaches the TS. Analysing geometrical parameters of TS leading to cycloadducts **3** and **4,** it can be noted that the advancement stage of new bonds depends on reagent nature and—to a smaller extent—on medium polarity.

**Fig. 3:**
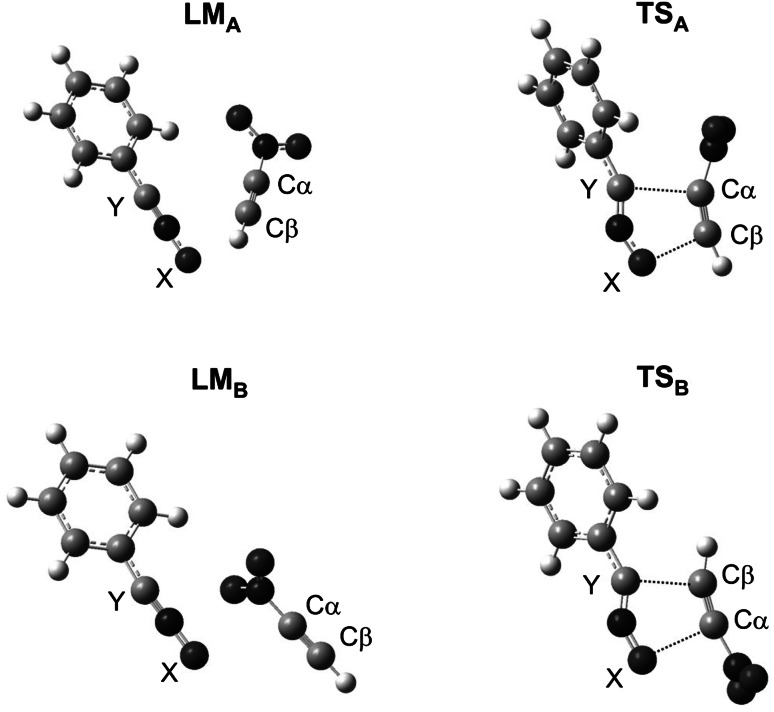
Key structures for reaction between nitroacetylene (**1**) and benzonitrile *N*-oxide (**2a**) in toluene according to DFT calculations at B3LYP/6-31G(d) theory level

So, in the case of a reaction with benzonitrile *N*-oxide in toluene, the bond at the β carbon atom introduced from nitroacetylene always is more advanced at the TS (Fig. 3). Formation of bonds in the area of the less favoured energetically TS_B_ takes place in a more asynchronous manner (∆*l* > 0.2). Both TSs have a polar nature, which is reflected in the value of GEDT index (see Table 2).

In case of a similar reaction with phenyl azide in the same reaction medium, both analysed TSs show a similar level of asynchronicity. The more energetically favourable TS_B_ is the more polar one of the two.

On the other hand, the nature of TSs of the reaction using phenyldiazomethane shows complete difference. In particular, the less energetically favourable TS_A_ is characterized by an almost ideal synchronicity of new σ bond formation and a very weak polar character. On the other hand, in the area of TS_B_ new σ bonds are formed in a strongly asynchronous manner, accompanied by a strong charge-transfer effect towards the dipolarophile substructure. The asynchronicity of this TS is the greatest among all considered critical structures.

With the introduction of a stronger polar medium of nitromethane into the reaction medium, key parameters of the analysed structures do not change significantly. In the area of most TSs, asynchronicity of formation of new σ bonds increases. However, it does not increase enough to enforce a change in reaction mechanism.

Within TSs on paths **C** and **D** only one bond is always formed. It is always the bond with the more nucleophilic atom of the X 1,3-dipole (Fig. 3). Progress of this bond is always greater than 50 % (see *l* values in Table 2). It should be noted that TSs on paths leading to zwitterions **7** and **8** are always more polar than competitive units on paths leading to cycloadducts **3** and **4**. Also in the case of these structures, the polarity increase of the reaction medium does not change their nature substantially. Only their polarity becomes more pronounced (see GEDT values In Table 2).

## Conclusion

DFT calculations for various theory levels show that [2 + 3] cycloaddition reactions of nitroacetylene with allenyl-type TACs take place according to the polar mechanism. This is not, however, the expected two-step, zwitterionic mechanism, but a one-step mechanism. Zwitterionic structures with the “extended” conformation may theoretically form along competitive paths. However, this route is supported by neither kinetic nor thermodynamic factors. Despite the clearly polar nature of the reactions discussed, the influence of the polarity of the reaction medium on their kinetics and the nature of critical structures is relatively small.

## Computational details

All calculations reported in this paper were performed on “Zeus” supercomputer in the “Cyfronet” computational centre in Cracow.

Global and local electronic properties of reactants were estimated according to the equations described earlier. In particular, the electronic chemical potentials (*μ*) and chemical hardness (*η*) were evaluated in terms of one-electron energies of FMO (*E*_HOMO_ and *E*_LUMO_) using the equations:

$$ \mu \approx (E_{\text{HOMO}} + E_{\text{LUMO}} )/2\quad \quad \eta \approx E_{\text{LUMO}} - E_{\text{HOMO}} . $$

Next, the values of *μ* and *η* were then used for the calculation of global electrophilicity (*ω*) [16, 17] according to the formula:

$$ \omega = \mu^{2} /2\eta $$

and the global nucleophilicity (*N*) [18] of TACs can be expressed in terms of equation:

$$ N = E_{{{\text{HOMO}}\; ( {\text{TAC)}}}} - E_{{{\text{HOMO}}\;\left( {\text{tetracyanoethene}} \right)}} . $$

The local electrophilicity (*ω*_k_) [19] condensed to atom k was calculated by projecting the index *ω* onto any reaction centre k in the molecule by using Parr function P_k_^+^ [20]:

$$ \omega_{\text{k}} = {\text{P}}^{ + }_{\text{k}} \cdot\omega . $$

The local nucleophilicity (*N*_k_) [21] condensed to atom k was calculated using global nucleophilicity *N* and Parr function P_k_^−^ [20] according to the formula:

$$ N_{\text{k}} = {\text{P}}^{ - }_{\text{k}} \cdot\,N. $$

Reactivity indexes calculated on this way are collected in Table 1.

For the simulation of the reaction paths hybrid functional B3LYP with the 6-31G(d), basis set included in the GAUSSIAN 09 package [22] was used. It was found previously that the B3LYP/6-31G(d) calculations illustrate well the structure of TSs in polar [2 + 3] cycloadditions involving conjugated nitroalkenes [14, 23, 24]. The critical points on reaction paths were localized in an analogous manner as in the case of the previously analysed [2 + 3] cycloadditions of (*Z*)-*C*,*N*-diphenylnitrone with *gem*-dinitroethene [14]. In particular, for structure optimization of the reactants and the reaction products the Berny algorithm was applied. First-order saddle points were localized using the QST2 procedure. The TSs were verified by diagonalization of the Hessian matrix and by analysis of the intrinsic reaction coordinates (IRC). In addition, similar simulations using more advanced B3LYP/6-31+G(d), B3LYP/6-311G(d), as well as B3LYP/6-311+G(d) theoretical levels were performed.

All calculations were carried out for the simulated presence of toluene or nitromethane as the reaction medium. For this purpose PCM model [25] was used. For optimized structures the thermochemical data for the temperature *T* = 298 K and pressure *p* = 1 atm were computed using vibrational analysis data. Global electron density transfer (GEDT) [26] was calculated according to the formula:

$$ GEDT = - \varSigma q_{\text{A}} , $$

where *q*_A_ is the net Mulliken charge and the sum is taken over all the atoms of dipolarophile.

Indexes of σ-bonds development (*l*) were calculated according to formula [27]:

$$ l_{\text{A - B}} = 1 - \frac{{r_{{{\text{A}}\text{ - }{\text{B}}}}^{\text{TS}} - r_{{{\text{A}}\text{ - }{\text{B}}}}^{\text{P}} }}{{r_{{{\text{A}}\text{ - }{\text{B}}}}^{\text{P}} }}, $$

where *r*_A−B_^TS^ is the distance between the reaction centres A and B at the TS and *r*_A−B_^P^ is the same distance at the corresponding product.

The kinetic parameters as well as essential properties of critical structures are displayed in Tables 2, 3, 4.

**Table 3:** Eyring parameters for reactions between nitroacetylene (**1**) and TACs **2a**-**2c** according to B3LYP/6-31G(d) calculations

1,3-Dipole	Transition	Toluene (*ε* = 2.38)			Nitromethane (*ε* = 38.20)		
		∆*H*/kJ mol^−1^	∆*G*/kJ mol^−1^	∆*S*/J mol^−1^ K^−1^	∆*H*/kJ mol^−1^	∆*G*/kJ mol^−1^	∆*S*/J mol^−1^ K^−1^
**2a**	**1** + **2a** → **LM**_**A**_	−13.8	21.8	−119.2	−7.1	26.4	−112.1
	**1** + **2a** → **TS**_**A**_	41.4	92.9	−172.4	45.6	95.0	−166.5
	**1** + **2a** → **3a**	−351.5	−287.4	−214.2	−347.7	−284.5	−212.1
	**1** + **2a** → **LM**_**B**_	−7.9	28.5	−122.2	−4.2	30.5	−117.2
	**1** + **2a** → **TS**_**B**_	46.0	95.8	−167.4	46.4	96.2	−166.9
	**1** + **2a** → **4a**	−348.9	−287.9	−204.6	−347.3	−278.2	−231.4
**2b**	**1** + **2b** → **LM**_**A**_	−2.9	24.3	−92.0	−1.7	24.3	−86.2
	**1** + **2b** → **TS**_**A**_	59.4	109.6	−168.2	61.5	111.7	−168.6
	**1** + **2b** → **3b**	−298.7	−238.1	−202.5	−299.6	−239.3	−202.1
	**1** + **2b** → **LM**_**B**_	−5.0	23.0	−94.6	−2.9	26.4	−98.3
	**1** + **2b** → **TS**_**B**_	59.0	108.4	−164.8	58.2	107.1	−164.8
	**1** + **2b** → **4b**	−327.2	−266.1	−205.4	−332.2	−271.5	−204.6
	**1** + **2b** → **LM**_**C**_	−5.4	21.8	−91.6	−2.9	23.0	−87.0
	**1** + **2b** → **TS**_**C**_	125.1	169.9	−150.2	111.3	157.7	−155.6
	**1** + **2b** → **7b**	72.8	120.5	−160.7	66.1	114.6	−163.2
	**1** + **2b** → **LM**_**D**_	−1.3	26.8	−94.1			
	**1** + **2b** → **TS**_**D**_	154.8	205.9	−171.1	152.7	205.0	−174.9
	**1** + **2b** → **8b**	136.0	186.6	−169.5	128.9	179.5	−169.9
**2c**	**1** + **2c** → **LM**_**A**_	−5.0	30.1	−118.4	−5.9	19.7	−85.4
	**1** + **2c** → **TS**_**A**_	37.7	88.3	−169.5	38.1	87.9	−167.4
	**1** + **2c** → **3c**	−231.0	−172.8	−194.6	−231.8	−172.4	−199.6
	**1** + **2c** → **TS**_**B**_	33.5	82.4	−164.4	28.5	77.0	−164.0
	**1** + **2c** → **3c**	−233.5	−176.1	−192.5	−238.1	−180.3	−193.7
	**1** + **2c** → **TS**_**C**_	63.6	108.4	−151.0	50.6	95.8	−151.9
	**1** + **2c** → **7c**	−9.2	41.0	−168.6	−17.2	32.6	−166.9
	**1** + **2c** → **TS**_**D**_	109.2	161.9	−176.6	97.9	150.6	−177.8
	**1** + **2c** → **8c**	66.9	115.9	−164.8	59.4	107.9	−162.3

**Table 4:** Eyring parameters for reactions between nitroacetylene (**1**) and benzonitrile *N*-oxide (**2a**) in toluene according to DFT calculations at B3LYP/6-31+G(d), B3LYP/6-311G(d), and B3LYP/6-311+G(d) theory levels

Theory level	Transition	∆*H*/kJ mol^−1^	∆*G*/kJ mol^−1^	∆*S*/J mol^−1^ K^−1^
B3LYP/6-31+G(d)	**1** + **2a** → **LM**_**A**_	−10.0	18.0	−93.3
	**1** + **2a** → **TS**_**A**_	48.5	95.8	−158.6
	**1** + **2a** → **3a**	−333.5	−270.7	−211.7
	**1** + **2a** → **LM**_**B**_	−2.9	32.6	−119.7
	**1** + **2a** → **TS**_**B**_	53.1	102.5	−165.3
	**1** + **2a** → **4a**	−332.2	−271.1	−204.2
B3LYP/6-311G(d)	**1** + **2a** → **LM**_**A**_	−11.7	19.7	−105.4
	**1** + **2a** → **TS**_**A**_	49.8	100.0	−168.2
	**1** + **2a** → **3a**	−319.7	−256.5	−211.3
	**1** + **2a** → **LM**_**B**_	−7.1	26.8	−112.5
	**1** + **2a** → **TS**_**B**_	55.6	104.6	−164.8
	**1** + **2a** → **4a**	−318.4	−257.7	−204.2
B3LYP/6-311+G(d)	**1** + **2a** → **LM**_**A**_	−10.0	17.6	−92.0
	**1** + **2a** → **TS**_**A**_	53.6	99.2	−154.0
	**1** + **2a** → **3a**	−310.9	−248.5	−209.2
	**1** + **2a** → **LM**_**B**_	−0.8	26.8	−92.5
	**1** + **2a** → **TS**_**B**_	59.0	107.5	−163.6
	**1** + **2a** → **4a**	−309.6	−248.1	−205.4

## References

[1] Rall KB, Vil’davskaya AI, Petrov AA (1975). Russ Chem Rev.

[2] Zhang O-Xi, Eaton PE, Steele I, Gilardib R (2002). Synthesis.

[3] Benchouk W, Mekelleche SM, Silvi B, Aurell MJ, Domingo LR (2011). J Phys Org Chem.

[4] Jawalekar AM, Reubsaet E, Rutjes FPJT, van Delft FL (2011). Chem Commun.

[5] Domingo LR, Chamorro E, Pérez P (2009). Eur J Org Chem.

[6] Domingo LR, Aurell MJ, Perez P (2014). Tetrahedron.

[7] Bekhradnia AR, Arshadi S, Siadati SA (2014). Chem Papers.

[8] Lopez SA, Munk ME, Houk KN (2013). J Org Chem.

[9] Goulioukina NS, Makukhin NN, Beletskaya IP (2011). Tetrahedron.

[10] Kurpet M, Jędrysiak R, Suwiński J (2013). Chem Heterocyclic Compd.

[11] Larina L, Lopyrev V (2009). Nitroazoles: Synthesis, Structure and Applications.

[12] Boyer JH (1986). Nitroazoles: the C-nitro derivatives of five-membered N- and N, O-heterocycles.

[13] Khlebnikov AF, Koneva AS, Virtseva AA, Yufit DS, Mlostoń G, Heimgartner H (2014). Helv Chim Acta.

[14] Jasiński R (2013). Tetrahedron.

[15] Jasiński R, Kwiatkowska M, Barański A (2007). Wiad Chem.

[16] Parr RG, von Szentpaly L, Liu LS (1999). J Am Chem Soc.

[17] Domingo LR, Aurell MJ, Perez P, Contreras R (2002). Tetrahedron.

[18] Domingo LR, Chamorro E, Perez P (2008). J Org Chem.

[19] Domingo LR, Aurell MJ, Perez P, Contreras R (2002). J Phys Chem A.

[20] Domingo LR, Perez P, Saez JA (2013). RSC Adv.

[21] Perez P, Domingo LR, Duque-Norena M, Chamorro E (2009). J Mol Struct (TheoChem).

[22] Frisch MJ, Trucks GW, Schlegel HB, Scuseria GE, Robb MA, Cheeseman JR, Scalmani G, Barone V, Mennucci B, Petersson GA, Nakatsuji H, Caricato M, Li X, Hratchian HP, Izmaylov AF, Bloino J, Zheng G, Sonnenberg JL, Hada M, Ehara M, Toyota K, Fukuda R, Hasegawa J, Ishida M, Nakajima T, Honda Y, Kitao O, Nakai H, Vreven T, Montgomery JA, Peralta JE, Ogliaro F, Bearpark M, Heyd JJ, Brothers E, Kudin KN, Staroverov VN, Kobayashi R, Normand J, Raghavachari K, Rendell A, Burant JC, Iyengar SS, Tomasi J, Cossi M, Rega N, Millam NJ, Klene M, Knox JE, Cross JB, Bakken V, Adamo C, Jaramillo J, Gomperts R, Stratmann RE, Yazyev O, Austin AJ, Cammi R, Pomelli C, Ochterski JW, Martin RL, Morokuma K, Zakrzewski VG, Voth GA, Salvador P, Dannenberg JJ, Dapprich S, Daniels AD, Farkas O, Foresman JB, Ortiz JV, Cioslowski J, Fox DJ (2009). Gaussian 09, Revision A.01.

[23] Jasiński R, Ziółkowska M, Demchuk OM, Maziarka A (2014). Central Eur J Chem.

[24] Jasiński R (2009). Coll Czech Chem Commun.

[25] Cossi M, Rega N, Scalmani G, Barone V (2003). J Comp Chem.

[26] Domingo LR (2014). RSC Adv.

[27] Jasiński R, Kwiatkowska M, Baranski R (2009). J Mol Struct (TheoChem).

